# Effects of Doxorubicin on Extracellular Matrix Regulation in Primary Cardiac Fibroblasts from Mice

**DOI:** 10.1186/s13104-023-06621-7

**Published:** 2023-11-16

**Authors:** Cameron Skaggs, Steve Nick, Conner Patricelli, Laura Bond, Kali Woods, Luke Woodbury, Julia Thom Oxford, Xinzhu Pu

**Affiliations:** 1 Biomolecular Research Center, Boise State University, Boise, ID 83725 USA; 2 Biomolecular Sciences Graduate Program, Boise State University, Boise, ID 83725 USA; 3 Department of Biological Sciences, Boise State University, Boise, ID 83725 USA

**Keywords:** Doxorubicin, Cardiotoxicity, Extracellular matrix, Cardiac fibroblasts, Proteomics

## Abstract

**Objective:**

Doxorubicin (DOX) is a highly effective chemotherapeutic used to treat many adult and pediatric cancers. However, its use is limited due to a dose-dependent cardiotoxicity, which can lead to lethal cardiomyopathy. In contrast to the extensive research efforts on toxic effects of DOX in cardiomyocytes, its effects and mechanisms on cardiac extracellular matrix (ECM) homeostasis and remodeling are poorly understood. In this study, we examined the potential effects of DOX on cardiac ECM to further our mechanistic understanding of DOX-induced cardiotoxicity.

**Results:**

DOX-induced significant down-regulation of several ECM related genes in primary cardiac fibroblasts, including Adamts1, Adamts5, Col4a1, Col4a2, Col5a1, Fbln1, Lama2, Mmp11, Mmp14, Postn, and TGF_β_. Quantitative proteomics analysis revealed significant global changes in the fibroblast proteome following DOX treatment. A pathway analysis using iPathwayGuide of the differentially expressed proteins revealed changes in a list of biological pathways that involve cell adhesion, cytotoxicity, and inflammation. An apparent increase in Picrosirius red staining indicated that DOX-induced an increase in collagen production in cardiac primary fibroblasts after 3-day treatment. No significant changes in collagen organization nor glycoprotein production were observed.

**Supplementary Information:**

The online version contains supplementary material available at 10.1186/s13104-023-06621-7.

## Introduction

Cancer treatment has improved significantly in recent years. However, the applicability of some anticancer drugs is limited by the risk of cardiotoxicity [1]. One classic example is DOX. DOX is a highly effective chemotherapeutic used to treat many adult and pediatric cancers, such as solid tumors, leukemia, lymphomas and breast cancer [2–4]. However, its use is limited due to a dose-dependent cardiotoxicity, which can lead to lethal cardiomyopathy [5–8]. While multiple mechanisms have been shown to be responsible for DOX-induced cardiotoxicity, it is generally accepted that the principle mechanism is oxidative stress induction through the production of reactive oxygen species (ROS) and free radicals in the myocardium [4, 6, 9, 10]. The increased level of oxidative stress can subsequently induce apoptosis and cell death in cardiomyocytes [3, 4, 11]. Efforts to reduce/prevent DOX-induced cardiotoxicity using antioxidants have largely failed in pre-clinical and clinical trials, indicating that oxidative stress may only partially explain the cardiotoxicity [9]. Thus, novel mechanisms responsible for DOX-induced cardiotoxicity and corresponding intervention measures need to be explored to expand the use of this effective anticancer drug.

The cellular constituents of the heart include cardiac fibroblasts, cardiomyocytes, endothelial cells, vascular smooth muscle cells, and transient cells such as leukocytes [12, 13]. Fibroblasts are the largest cell population in the heart and play a critical role in normal cardiac function [12]. The toxic effects of DOX in cardiomyocytes have been extensively investigated [5, 14, 15]. In contrast, data on the effects and mechanisms of these drugs on cardiac fibroblasts and ECM homeostasis is limited. In this study, we examined the potential short-term effects of DOX on cardiac fibroblasts to further our understanding of the mechanisms of DOX-induced cardiotoxicity, which may lead to novel intervention measures to improve the therapeutic options for cancer treatment.

## Methods

### Cell culture

Cardiac fibroblasts from BALB/c mice were obtained from Cell Biologics (Chicago, IL, USA) and cultured in fibroblast medium provided by the vendor, which contained fibroblasts growth factor, hydrocortisone, antibiotics–antimycotics, 2 mM l-glutamine, and 10% fetal bovine serum. Cells were maintained at 37 °C with 5% CO_2_.

### ECM Gene Expression Profiling

Cardiac fibroblasts were seeded at a density of 2 × 10^5^ cells per well in 6-well plates. After overnight incubation, the cells were treated with 1 μM DOX or a vehicle control for 24 h. The selection of 1 μM DOX concentration was based on previous cell viability results (unpublished data) in our lab that showed approximately 70% cell viability after 24-h treatment. At the end of the treatment, cells were harvested. Total RNA was extracted from the cells using an RNeasy Mini Kit (Qiagen, Germantown, MD, USA). The expression of ECM related genes was analyzed using a Mouse Extracellular Matrix and Adhesion Molecules RT^2^ Profiler PCR Array (PAMM-013ZA, Qiagen, Germantown, MD, USA) following the manufacturer’s instructions. Briefly, 100 ng RNA from each sample was reverse transcribed into cDNA using a RT^2^ first strand kit. Twenty five µL of cDNA was then mixed with SYBR Green mastermix. Real-time PCR was performed on a LightCycler® 96 (Roche Diagnostics Corporation, Indianapolis, IN, USA). A web-based tool from Qiagen, RT^2^ Profiler PCR Data Analysis, was used for differential gene expression analysis.

### LC–MS based quantitative proteomics analysis

A liquid chromatography-mass spectrometry (LC–MS) based quantitative proteomics approach was used to assess the relative protein expression in cardiac fibroblasts following DOX treatment. A Tandem Mass Tag (TMT) labeling assay was used for LC–MS based protein quantification (Additional file 1: Figure S1). Briefly, cardiac fibroblasts were seeded at a density of 5 × 10^5^ cells per flask in T25 cell culture flasks. After overnight incubation, the cells were treated with 1 μM DOX or a vehicle control for 24 h. Cellular proteins were extracted using radioimmunoprecipitation assay (RIPA) buffer containing protease and phosphatase inhibitors.

TMT labeling was performed using a reagent kit from ThermoFisher Scientific (Waltham, MA, USA). Protein sample preparation, including reduction, alkylation, tryptic digestion, and TMT labeling was performed following the manufacturer’s instructions. The resulting labeled peptide mixtures were fractionated using a Pierce™ high pH reversed-phase peptide fractionation kit (ThermoFisher Scientific, Waltham, MA, USA). Each fraction was then dried under vacuum and reconstituted in 5% acetonitrile and 0.1% formic acid. LC–MS analysis of the labeled peptides was performed on a Velos Pro Dual-Pressure Linear Ion Trap mass spectrometer equipped with a nano electrospray ionization source and coupled with an Easy-nLC II nano LC system (Thermo Fisher Scientific, Waltham, MA, USA). Peptide spectral matching and protein identification were achieved by database search using Sequest HT algorithms in Proteome Discoverer 2.2 (Thermo Fisher Scientific, Waltham, MA, USA). Raw spectrum data were searched against the UniProtKB/Swiss-Prot protein database for mouse (downloaded from www.uniprot.org on 9/8/2022). A decoy database search was performed to calculate false discovery rate (FDR). Proteins containing two or more peptides with FDR ≤ 0.01were considered positively identified. Protein quantification and differential analysis were performed using Proteome Discoverer 2.2 and MSstatsTMT, an R package for statistical analysis of quantitative mass spectrometry-based proteomic experiments (Additional file 1: Additional method) [16]. Differentially expressed proteins were further analyzed using iPathwayGuide (iPG; Advaita Bioinformatics, Ann Arbor, MI, USA) to identify significantly impacted pathways in the fibroblasts [17].

### Extracellular matrix staining

Picrosirius red and alcian blue staining were used to examine the effects of DOX on the production and structure of ECM in cardiac fibroblasts. Cardiac fibroblasts were seeded at 2 × 10^4^ per well in a 6-well plate, incubated overnight, and treated with 1 µM DOX for 72 h. Cells were then washed with cold PBS and fixed with cold methanol. For alcian blue staining, cells were acidified with 3% acetic acid for 3 min and stained with alcian blue for 30 min. For Picrosirius red staining, fixed cells were incubated with 0.1% Sirius red in saturated picric acid for one hours. Cells from both staining procedures were washed with PBS and observed under a light microscope.

## Results

### ECM Gene Expression Profile

**Fig. 1:**
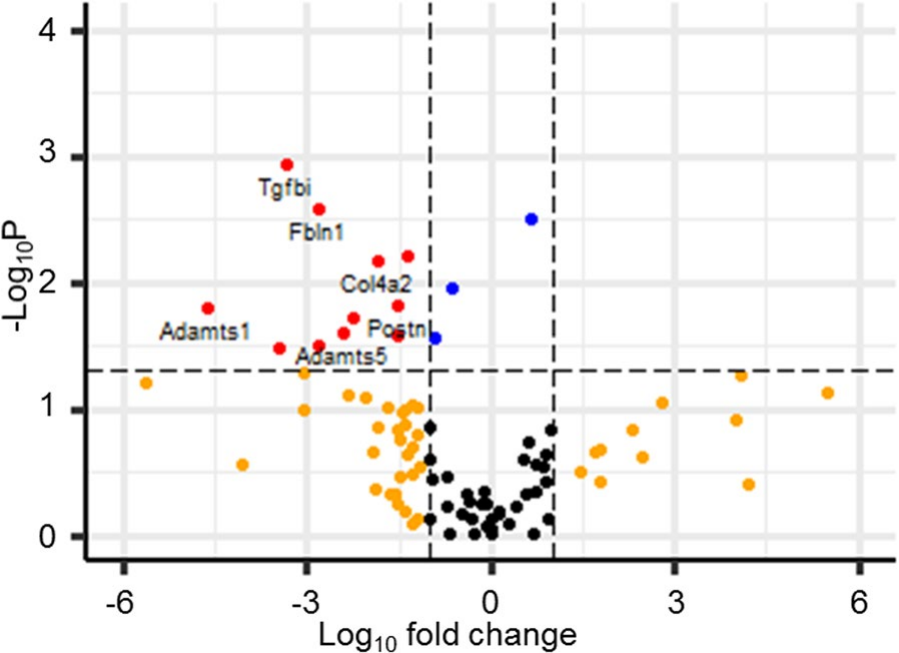
Effects of DOX on the expression of the ECM and adhesion genes in primary cardiac fibroblasts isolated from BALB/c mice. Cells were treated with 1 µM DOX for 24 h. Experiments were performed in triplicates. Student’s t-test with Bonferroni correction was used for statistical analysis

We used a Mouse Extracellular Matrix and Adhesion Molecules RT2 Profiler PCR Array obtained from Qiagen to examine the effect of DOX on the expression of the ECM related genes in primary cardiac fibroblasts. Several genes were significantly downregulated after cells were treated with 1 µM DOX for 24 h (Fig. 1). These genes include Adamts1, Adamts5, Col4a1, Col4a2, Col5a1, Fbln1, Lama2, Mmp11, Mmp14, Postn, and TGF_β_. These results indicate that DOX treatment induced an interference with the expression of genes that are involved in the maintenance of ECM homeostasis in cardiac fibroblasts.

### Quantitative Proteomics Analysis

A LC–MS based quantitative proteomics analysis was implemented to examine the global changes in the proteome in primary cardiac fibroblast following DOX treatment. The results of this experiment show that DOX induced changes in the expression of several proteins in the cardiac fibroblasts after 24-h treatment (Table 1). A pathway analysis using iPG of the differentially expressed proteins revealed changes in a list of biological pathways in the cells that involve cell adhesion, cytotoxicity, and inflammation response (Additional file 1: Table S1).

**Table 1:** Differentially expressed proteins in primary cardiac fibroblasts isolated from BALB/c mice

Uniprot Accession #	Protein Description	Fold of Change
*Up regulated*		
Q64695	Endothelial protein C receptor	3.3
P13597-2	Isoform 2 of Intercellular adhesion molecule 1	2.5
Q9JHW9	Aldehyde dehydrogenase family 1 member A3	2.4
P48999	Arachidonate 5-lipoxygenase	2.0
P16125	L-lactate dehydrogenase B chain	2.0
Q9CRC6	BLOC-1-related complex subunit 7	1.9
Q62433	Protein NDRG1	1.8
Q5I2A0	Serine protease inhibitor A3G	1.8
O35484	Antizyme inhibitor 1	1.8
*Down regulated*		
O35988	Syndecan-4	2.5
Q3TLR7	Denticleless protein homolog	2.4
Q9DAD6	Profilin-3	2.0
Q7TPV4	Myb-binding protein 1A	2.0
Q9WTW3	Potassium voltage-gated channel subfamily E member 4	1.9
P35441	Thrombospondin-1	1.9
Q9DAM7	Transmembrane protein 263	1.8
Q8BVY0	Ribosomal L1 domain-containing protein 1	1.8
Q03350	Thrombospondin-2	1.8
P29268	Connective tissue growth factor	1.8

Cells were treated with 1 µM DOX for 24 h (n = 5; p < 0.05)

Relative protein quantification was determined using a LC–MS based quantitative proteomics approach

### Extracellular matrix staining

**Fig. 2:**
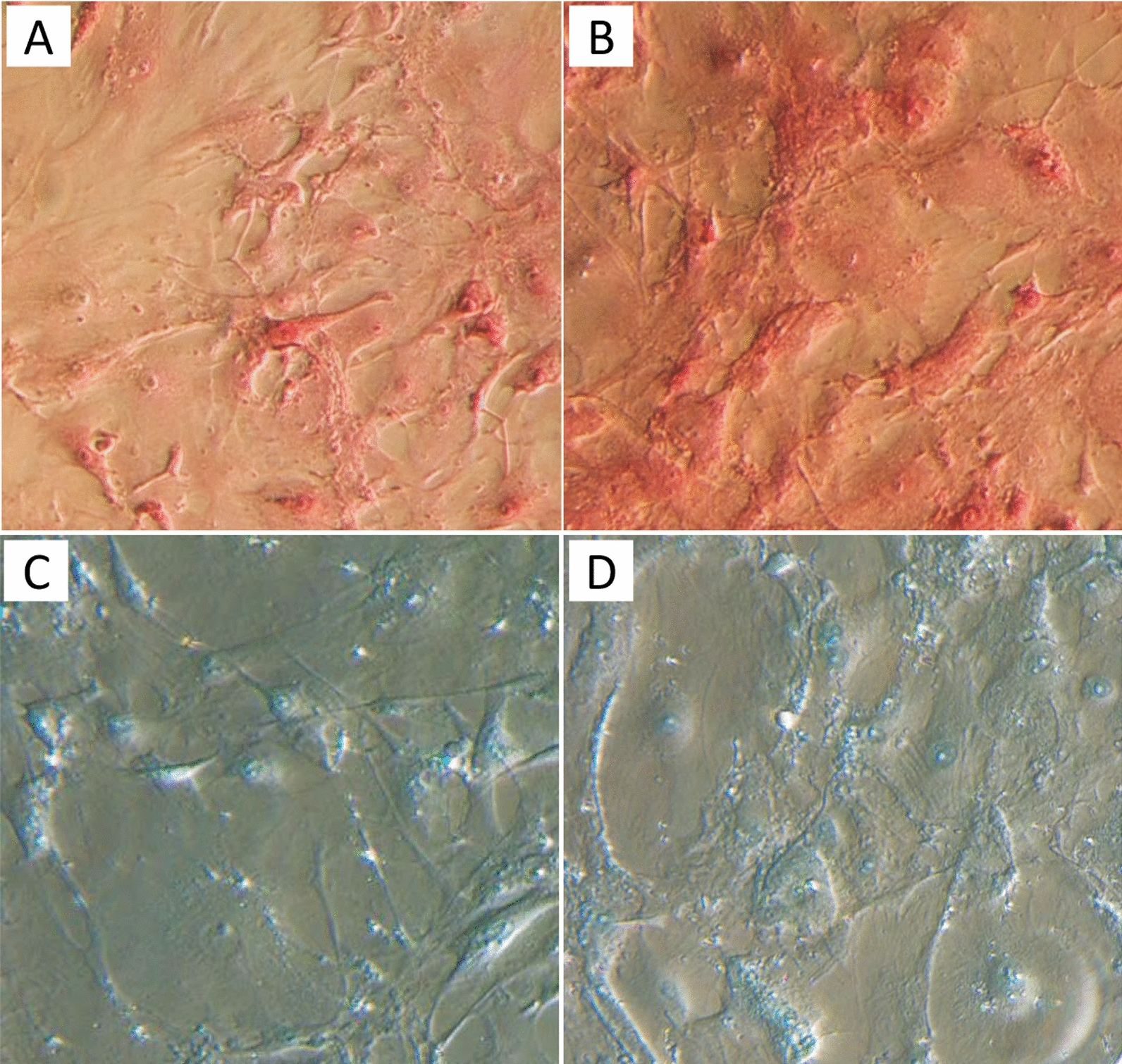
Extracellular matrix staining of primary cardiac fibroblasts isolated from BALB/c mice treated with 1 µM DOX for 72 h. **A** Picrosirius Red staining, control cells; **B** Picrosirius Red staining, DOX treated cells; **C** Alcian blue staining, control cells; **D** Alcian blue staining, DOX treated cells

Cardiac fibroblasts were stained with Picrosirius red to examine the effects of DOX on the production and structural organization of collagens. No significant changes in collagen organization were observed (Fig. 2 A, B). However, an apparent increase in Picrosirius red staining indicates that DOX induced an increase in collagen production in cardiac primary fibroblasts after 3-day treatment at 1 µM concentration (Fig. 2 A, B). No significant changes were observed in alcian blue staining for glycoproteins (Fig. 2 C, D).

## Discussion

DOX, a member of the anthracycline family, has been one of the most widely used anticancer drugs since it was first approved by the U.S Food and drug Administration (FDA) for clinical use [18]. It is well documented that this drug induces a dose-related cardiotoxicity, which is one of the most common etiologies of cancer chemotherapy-associated heart failure [18–20]. Toxicity of DOX in cardiomyocytes has been extensively studied in the last several decades, and multiple mechanisms have been proposed, including oxidative stress, topoisomerase inhibition, ferroptosis, cardiogenetics, mitochondrial bio-energetics, etc. [18, 21–23]. In this study, we investigated the potential adverse effects of DOX on a different major cellular constituent of the heart, cardiac fibroblasts.

Cardiac fibroblasts are the main cell type responsible for the synthesis, deposition, and degradation of cardiac extracellular matrix (ECM) [12]. Cardiac ECM not only provides structural support for cardiac cells, but also plays important roles in electrical signaling, secretion of growth factors and cytokines, and potentiating blood vessel formation [12]. There were limited reports indicating DOX may affect fibroblast function and the homeostasis of cardiac ECM, but the mechanisms and impacts on cardiomyocyte functions are not clear [24–30]. Our results demonstrated a significant alteration in the expression of ECM related genes associated with maintenance, structural organization and remolding when exposed to DOX. We found that DOX induced the downregulation of Adamts1, Adamts5, Col4a1, Col4a2, Col5a1, Fbln1, Lama2, Mmp11, Mmp14, Postn, and TGF_β_. These genes play important roles in structural organization and remodeling of ECM. Col4a1 and Col4a2 encode the collagen IV protein α1 and α2 chains, respectively [31]. Collagen IV is the major structural component of the basement membrane and is essential for its integrity and functionality [32–34]. Disruption of collagen IV has been linked to cardiovascular diseases [31, 33, 35]. Col5a has been found to regulate wound healing and scar size after heart injury [36]. Depletion of collagen V led to enhanced myofibroblast differentiation and increased post-infarction scar size with worsening of heart function [36]. ADAMTS (a disintegrin and metalloproteinase with thrombospondin motifs) is a family of 19 proteases with diverse functions, including the processing of collagen, cleavage of matrix proteoglycans, and proteolysis of von Willebrand factor [37–39]. ADAMTSs have been shown to play multiple distinct roles in cardiovascular tissues [38]. As the major ECM-degrading enzymes, matrix metalloproteinases (MMPs) have been a focus of cardiovascular research for decades [40, 41]. MMPs have been associated with many cardiovascular conditions, including atherosclerosis, coronary artery disease, myocardial infarction, and heart failure [41, 42]. Transforming growth factor beta (TGF_β_) regulates the phenotype and function of cardiomyocytes, fibroblasts, immune cells and vascular cells, and plays a major role in cardiac fibrosis [43, 44]. Three of the additional genes downregulated by DOX in this study, Lama2, Postn, and Fbln1, are also important factors in cardiovascular ECM remodeling [45–47]. Clinical observation and previous experimental studies indicate that DOX treatment impairs wound healing [24–27], reduces collagen production, and inhibits skin fibroblast proliferation [28]. DOX was also found to increase matrix metalloprotease 9 (MMP9) expression [29] and cause chronic fibrosis in the myocardium [30].

Quantitative proteomics analysis in this study revealed that DOX induced certain global changes in the cardiac fibroblast proteome. Three of the differentially expressed proteins, syndecan-4, thrombospondins, and cellular communication network factor (CCN2), are known to play import roles in ECM regulation [48–50]. Additional studies in our lab confirmed via Western blot that DOX induced a dose-dependent decrease in TGF_β_-stimulated CCN2 expression in primary cardiac fibroblasts isolated from BALB/c mice (unpublished data).

Taken together, this study revealed evidence that DOX can modulate the expression of ECM genes in cardiac fibroblasts, which may affect the structure and functions of heart ECM. These results provided new insights to understand the mechanisms of DOX cardiotoxicity, which may lead to novel intervention measures to improve the therapeutic options for cancer treatment.

## Limitations

The expression of ECM related genes was analyzed using a pre-made Mouse Extracellular Matrix and Adhesion Molecules RT2 Profiler PCR Array (PAMM-013ZA, Qiagen, Germantown, MD, USA). Although this array covers 84 important genes (see the complete gene list in Additional file 1: Table S2) that are known to associate with ECM and cell adhesion, many more genes are involved in ECM production, regulation, and remodeling. The effects of DOX on genes that are not on this array need to be considered in future studies. This study was designed to induce an acute response and examine early events in cardiac fibroblasts. Multiple doses and time-points are needed to assess mRNA and protein changes to further elucidate changes in ECM remodeling. In addition, confirmation of protein level changes found in LC–MS experiment will need to be confirmed with Western blotting or similar methodologies.

### Supplementary Information

**Additional file 1: Additional Method.** R code for MSstatsTMT. **Figure S1.** LC-MS based quantitative proteomics workflow. A duplex TMT labeling kit was used for peptide labeling. **Table S1.** Significantly affected biological pathways in primary cardiac fibroblasts isolated from BALB/c mice treated with DOX. **Table S2.** Gene list for Qiagen Mouse ECM and Adhesion Molecules RT2 Profiler PCR Array

## Data Availability

The datasets used and/or analyzed during the current study are available from corresponding author upon request.

## Abbreviations

ADAMTS: A disintegrin and metalloproteinase with thrombospondin motifs
Col4a: Collagen, type IV
CCN2: Cellular communication network factor 2
DOX: Doxorubicin
ECM: Extracellular matrix
IACUC: Institutional Animal Care and Use Committee
iPG: iPathwayGuide
Lama2: Laminin, alpha 2
LC–MS: Liquid chromatography-Mass spectrometry
MMP: Matrix metalloproteinase
Postn: Periostin
TMT: Tandem mass tag
