# Supplementary material for: Effects of Doxorubicin on Extracellular Matrix Regulation in Primary Cardiac Fibroblasts from Mice
Source: BMC Res Notes. 2023 Nov 16;16:340. doi: 10.1186/s13104-023-06621-7 (PMC10655342; doi:10.1186/s13104-023-06621-7)
Supplement: Supplementary file 1 — Additional file 1: Additional Method. R code for MSstatsTMT. Figure S1. LC-MS based quantitative proteomics workflow. A duplex TMT labeling kit was used for peptide labeling. Table S1. Significantly affected biological pathways in primary cardiac fibroblasts isolated from BALB/c mice treated with DOX. Table S2. Gene list for Qiagen Mouse ECM and Adhesion Molecules RT2 Profiler PCR Array [file 13104_2023_6621_MOESM1_ESM.docx]

**Effects of Doxorubicin on Extracellular Matrix Regulation in Primary Cardiac Fibroblasts from Mice**

Cameron Skaggs^1^ , Steve Nick^1^, Conner Patricelli^1,2^, Laura Bond^1^, Kali Woods^1^, Luke Woodbury^1,2^, Julia Thom Oxford^1,2,3^, Xinzhu Pu^1,2,3^*

Additional file 1: Additional Method

R code for MSstatsTMT

require(MSstatsTMT)

require(tidyverse)

require(readxl)

require(limma)

require(readxl)

# For the MSStatsTMT workflow employed here, the field names should be

# consistent with required names for MSStatsTMT

# (https://www.bioconductor.org/packages/devel/bioc/vignettes/MSstatsTMT/inst/doc/MSstatsTMT.html)

# so that renaming will likely be required.

#

# Likewise an annotation file will also be required

# annotationDox.csv is used here

#

# check for mistakes

setdiff(unique(raw.input$Spectrum.File), annot.pd$Run)

setdiff(annot.pd$Run, unique(raw.input$Spectrum.File))

length(unique(raw.input$Protein.Accessions))

length(unique(raw.input$Master.Protein.Accessions))

# The value posted here needs to match the number of records in the annotation file

annot.pd %>%

summarize(uniqueBioReps = n_distinct(BioReplicate))

# The order of the channel fields in the raw.input file dictates the sort order of

# the channels in the annotation file

# You have to examine the order in the raw.input file

#arranging the annotation file based on channel (Treatment), mixture (replicate)

annot.pd <- annot.pd %>%

arrange(Channel, Mixture, Run, BioReplicate)

####End####

#Determine which Proteins are only in a single mixture for removal

SingleMix <- raw.input %>%

group_by(Master.Protein.Accessions,

Mixture)%>%

summarize(nCopies = n()) %>%

group_by(Master.Protein.Accessions) %>%

summarise(N = n()) %>%

filter(N == 1)

#removes the proteins in a single mixture from raw.input

raw.input<-raw.input%>%filter(!Master.Protein.Accessions %in% SingleMix$Master.Protein.Accessions)

#Runs proteins summarization. Using Master.Protein.Accessions else we lose our protein of interest.

input.pd <- PDtoMSstatsTMTFormat(raw.input, annotation = annot.pd,

which.proteinid = "Master.Protein.Accessions",

useUniquePeptide = FALSE,

rmPSM_withfewMea_withinRun = FALSE)

raw.input %>%

mutate(PSM = paste(Sequence,Charge,sep = "_")) %>%

group_by(PSM) %>%

summarize(nProteins = n_distinct(Master.Protein.Accessions)) %>%

filter(nProteins > 1) %>%

summarize(nRows = n())

input.pd$ProteinName <- as.character(input.pd$Protein)

input.pd$TechRepMixture<-as.character(input.pd$TechRepMixture)

input.pd$PeptideSequence<-as.character(input.pd$PeptideSequence)

input.pd$Run <- as.character(input.pd$Run)

input.pd$PSM<-as.character(input.pd$PSM)

input.pd$Channel<-as.character(input.pd$Channel)

input.pd$Mixture<-as.character(input.pd$Mixture)

input.pd$Condition<-as.character(input.pd$Condition)

input.pd$BioReplicate<-as.character(input.pd$BioReplicate)

input.pd$Charge<-as.character(input.pd$Charge)

#Default is msstats, summarizes the proteins

quant.pd.nonorm <- proteinSummarization(data = input.pd,

method = "msstats",

global_norm = TRUE, #global median normalization on peptide level data Default=TRUE

reference_norm=FALSE, #Reference channel based normalization between MS runs on a protein level data

MBimpute = TRUE)

levels(quant.pd.nonorm$Condition)

compDoxCtrl <- matrix(c(-1,1), nrow = 1)

row.names(compDoxCtrl) <- c("Dox-Control")

colnames(compDoxCtrl) <- c("control", "dox")

#performs the group comparison on the data

CompGroup <- groupComparisonTMT(data = quant.pd.nonorm,

contrast.matrix = compDoxCtrl,

moderated = TRUE,

adj.method = "fdr")

comps <- as.data.frame(CompGroup$ComparisonResult)

**
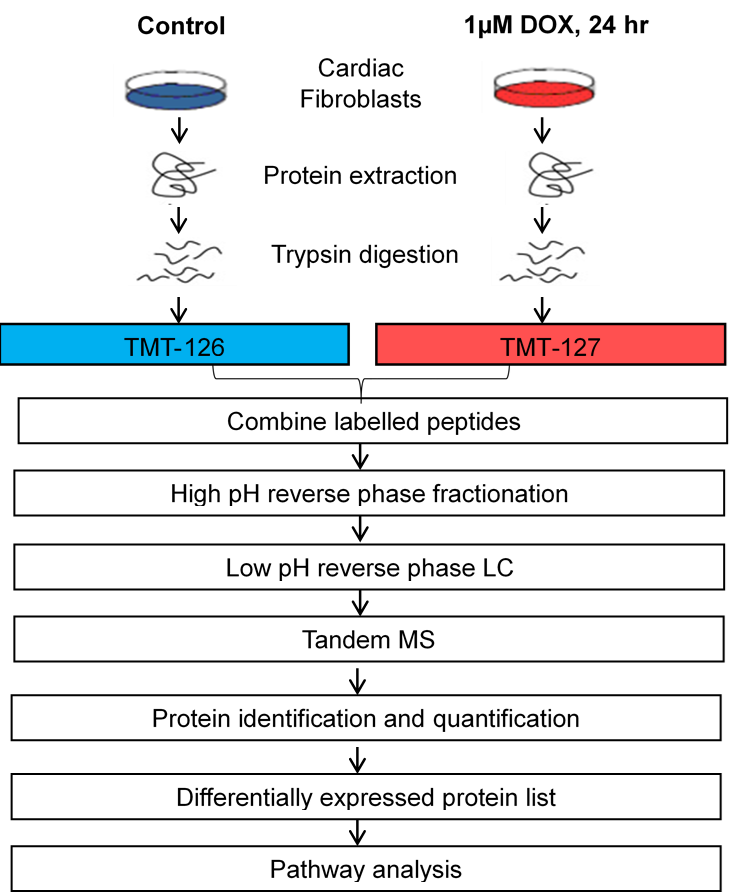
**

Additional file 1: Figure S1. LC-MS based quantitative proteomics workflow. A duplex TMT labeling kit was used for peptide labeling.

Additional file 1: Table S1. Significantly affected biological pathways in primary cardiac fibroblasts isolated from BALB/c mice treated with DOX.

| **Pathway Name** | **p-value** |
| --- | --- |
| Cell adhesion molecules (CAMs) | 0.000 |
| Staphylococcus aureus infection | 0.009 |
| African trypanosomiasis | 0.014 |
| Malaria | 0.015 |
| Natural killer cell mediated cytotoxicity | 0.016 |
| Fluid shear stress and atherosclerosis | 0.026 |
| Hippo signaling pathway | 0.027 |
| Rheumatoid arthritis | 0.031 |
| NF-kappa B signaling pathway | 0.031 |
| Viral myocarditis | 0.034 |
| Propanoate metabolism | 0.034 |
| ErbB signaling pathway | 0.038 |
| Cysteine and methionine metabolism | 0.04 |
| Pyruvate metabolism | 0.047 |

Additional file 1: Table S2. Gene list for Qiagen Mouse ECM and Adhesion Molecules RT2 Profiler PCR Array

| **UniGene** | **GenBank** | **Symbol** | **Description** |
| --- | --- | --- | --- |
| Mm.423621 | NM_009851 | Cd44 | CD44 antigen |
| Mm.35605 | NM_009864 | Cdh1 | Cadherin 1 |
| Mm.257437 | NM_007664 | Cdh2 | Cadherin 2 |
| Mm.4658 | NM_001037809 | Cdh3 | Cadherin 3 |
| Mm.184711 | NM_009867 | Cdh4 | Cadherin 4 |
| Mm.470343 | NM_007727 | Cntn1 | Contactin 1 |
| Mm.277735 | NM_007742 | Col1a1 | Collagen, type I, alpha 1 |
| Mm.2423 | NM_031163 | Col2a1 | Collagen, type II, alpha 1 |
| Mm.249555 | NM_009930 | Col3a1 | Collagen, type III, alpha 1 |
| Mm.738 | NM_009931 | Col4a1 | Collagen, type IV, alpha 1 |
| Mm.181021 | NM_009932 | Col4a2 | Collagen, type IV, alpha 2 |
| Mm.389135 | NM_007734 | Col4a3 | Collagen, type IV, alpha 3 |
| Mm.7281 | NM_015734 | Col5a1 | Collagen, type V, alpha 1 |
| Mm.2509 | NM_009933 | Col6a1 | Collagen, type VI, alpha 1 |
| Mm.390287 | NM_010217 | Ctgf | Connective tissue growth factor |
| Mm.18962 | NM_009818 | Ctnna1 | Catenin (cadherin associated protein), alpha 1 |
| Mm.34637 | NM_009819 | Ctnna2 | Catenin (cadherin associated protein), alpha 2 |
| Mm.291928 | NM_007614 | Ctnnb1 | Catenin (cadherin associated protein), beta 1 |
| Mm.3433 | NM_007899 | Ecm1 | Extracellular matrix protein 1 |
| Mm.286375 | NM_133918 | Emilin1 | Elastin microfibril interfacer 1 |
| Mm.2824 | NM_009848 | Entpd1 | Ectonucleoside triphosphate diphosphohydrolase 1 |
| Mm.297992 | NM_010180 | Fbln1 | Fibulin 1 |
| Mm.193099 | NM_010233 | Fn1 | Fibronectin 1 |
| Mm.266790 | NM_013500 | Hapln1 | Hyaluronan and proteoglycan link protein 1 |
| Mm.2168 | NM_010406 | Hc | Hemolytic complement |
| Mm.435508 | NM_010493 | Icam1 | Intercellular adhesion molecule 1 |
| Mm.5007 | NM_008396 | Itga2 | Integrin alpha 2 |
| Mm.57035 | NM_013565 | Itga3 | Integrin alpha 3 |
| Mm.31903 | NM_010576 | Itga4 | Integrin alpha 4 |
| Mm.16234 | NM_010577 | Itga5 | Integrin alpha 5 (fibronectin receptor alpha) |
| Mm.96 | NM_008399 | Itgae | Integrin alpha E, epithelial-associated |
| Mm.1618 | NM_008400 | Itgal | Integrin alpha L |
| Mm.262106 | NM_008401 | Itgam | Integrin alpha M |
| Mm.227 | NM_008402 | Itgav | Integrin alpha V |
| Mm.22378 | NM_021334 | Itgax | Integrin alpha X |
| Mm.263396 | NM_010578 | Itgb1 | Integrin beta 1 (fibronectin receptor beta) |
| Mm.1137 | NM_008404 | Itgb2 | Integrin beta 2 |
| Mm.87150 | NM_016780 | Itgb3 | Integrin beta 3 |
| Mm.213873 | NM_001005608 | Itgb4 | Integrin beta 4 |
| Mm.303386 | NM_008480 | Lama1 | Laminin, alpha 1 |
| Mm.256087 | NM_008481 | Lama2 | Laminin, alpha 2 |
| Mm.42012 | NM_010680 | Lama3 | Laminin, alpha 3 |
| Mm.425599 | NM_008483 | Lamb2 | Laminin, beta 2 |
| Mm.435441 | NM_008484 | Lamb3 | Laminin, beta 3 |
| Mm.1249 | NM_010683 | Lamc1 | Laminin, gamma 1 |
| Mm.14126 | NM_019471 | Mmp10 | Matrix metallopeptidase 10 |
| Mm.4561 | NM_008606 | Mmp11 | Matrix metallopeptidase 11 |
| Mm.2055 | NM_008605 | Mmp12 | Matrix metallopeptidase 12 |
| Mm.5022 | NM_008607 | Mmp13 | Matrix metallopeptidase 13 |
| Mm.280175 | NM_008608 | Mmp14 | Matrix metallopeptidase 14 (membrane-inserted) |
| Mm.217116 | NM_008609 | Mmp15 | Matrix metallopeptidase 15 |
| Mm.156952 | NM_032006 | Mmp1a | Matrix metallopeptidase 1a (interstitial collagenase) |
| Mm.29564 | NM_008610 | Mmp2 | Matrix metallopeptidase 2 |
| Mm.4993 | NM_010809 | Mmp3 | Matrix metallopeptidase 3 |
| Mm.4825 | NM_010810 | Mmp7 | Matrix metallopeptidase 7 |
| Mm.16415 | NM_008611 | Mmp8 | Matrix metallopeptidase 8 |
| Mm.4406 | NM_013599 | Mmp9 | Matrix metallopeptidase 9 |
| Mm.4974 | NM_010875 | Ncam1 | Neural cell adhesion molecule 1 |
| Mm.433941 | NM_010954 | Ncam2 | Neural cell adhesion molecule 2 |
| Mm.343951 | NM_008816 | Pecam1 | Platelet/endothelial cell adhesion molecule 1 |
| Mm.236067 | NM_015784 | Postn | Periostin, osteoblast specific factor |
| Mm.5245 | NM_011345 | Sele | Selectin, endothelial cell |
| Mm.1461 | NM_011346 | Sell | Selectin, lymphocyte |
| Mm.3337 | NM_011347 | Selp | Selectin, platelet |
| Mm.8739 | NM_011360 | Sgce | Sarcoglycan, epsilon |
| Mm.291442 | NM_009242 | Sparc | Secreted acidic cysteine rich glycoprotein |
| Mm.379020 | NM_009262 | Spock1 | Sparc/osteonectin, cwcv and kazal-like domains proteoglycan 1 |
| Mm.288474 | NM_009263 | Spp1 | Secreted phosphoprotein 1 |
| Mm.289702 | NM_009306 | Syt1 | Synaptotagmin I |
| Mm.14455 | NM_009369 | Tgfbi | Transforming growth factor, beta induced |
| Mm.4159 | NM_011580 | Thbs1 | Thrombospondin 1 |
| Mm.26688 | NM_011581 | Thbs2 | Thrombospondin 2 |
| Mm.2114 | NM_013691 | Thbs3 | Thrombospondin 3 |
| Mm.8245 | NM_011593 | Timp1 | Tissue inhibitor of metalloproteinase 1 |
| Mm.206505 | NM_011594 | Timp2 | Tissue inhibitor of metalloproteinase 2 |
| Mm.4871 | NM_011595 | Timp3 | Tissue inhibitor of metalloproteinase 3 |
| Mm.454219 | NM_011607 | Tnc | Tenascin C |
| Mm.76649 | NM_011693 | Vcam1 | Vascular cell adhesion molecule 1 |
| Mm.158700 | NM_001081249 | Vcan | Versican |
| Mm.3667 | NM_011707 | Vtn | Vitronectin |
| Mm.328431 | NM_007393 | Actb | Actin, beta |
| Mm.163 | NM_009735 | B2m | Beta-2 microglobulin |
| Mm.343110 | NM_008084 | Gapdh | Glyceraldehyde-3-phosphate dehydrogenase |
| Mm.3317 | NM_010368 | Gusb | Glucuronidase, beta |
| Mm.2180 | NM_008302 | Hsp90ab1 | Heat shock protein 90 alpha (cytosolic), class B member 1 |
